# Complete Genome Sequences of Four Enterohemolysin-Positive (*ehxA*) Enterocyte Effacement-Negative Shiga Toxin-Producing *Escherichia coli* Strains

**DOI:** 10.1128/genomeA.00846-16

**Published:** 2016-09-01

**Authors:** Sandra C. Lorenz, Michael L. Kotewicz, Maria Hoffmann, Narjol Gonzalez-Escalona, Markus Fischer, Julie A. Kase

**Affiliations:** a U.S. Food and Drug Administration, Center for Food Safety and Applied Nutrition, Division of Microbiology, College Park, Maryland, USA; b University of Hamburg, Hamburg School of Food Science, Institute of Food Chemistry, Hamburg, Germany; c U.S. Food and Drug Administration, Center for Food Safety and Applied Nutrition, Division of Molecular Biology, Laurel, Maryland, USA

## Abstract

Shiga toxin-producing *Escherichia coli* (STEC) strains are important foodborne pathogens associated with human disease. Most disease-associated STEC strains carry the locus of enterocyte effacement (LEE); however, regularly LEE-negative STEC strains are recovered from ill patients. Few reference sequences are available for these isolate types. Here, we report here the complete genome sequences for four LEE-negative STEC strains.

## GENOME ANNOUNCEMENT

Shiga toxin-producing *Escherichia coli* (STEC) strains are major foodborne pathogens that can cause mild/bloody diarrhea and life-threatening hemolytic-uremic syndrome (HUS). To date, hundreds of STEC serotypes have been implicated in human disease, with O157:H7 being the most predominant (1). While many STEC strains associated with severe disease possess the locus of enterocyte effacement (LEE) pathogenicity island and often harbor a large EHEC virulence plasmid carrying the enterohemolysin-encoding gene, *ehxA* (2–4), sporadically, LEE-negative STEC strains are recovered from severely ill individuals (5, 6). However, only a few complete reference sequences are available for those STEC strains; thus, we sequenced the complete genomes of four *ehxA*-positive LEE-negative STEC strains isolated from foods.

Genomic DNA from each strain was isolated from overnight cultures, according to the DNeasy blood and tissue kit (Qiagen, Inc., Valencia, CA) instructions, and the DNA templates were sheared to ≥10 kbp utilizing g-TUBEs (Covaris, Inc., Woburn, MA). Genomic libraries were prepared according to the PacBio 10-kbp insert library protocol using the DNA template prep kit 1.0 and were additionally size-selected with the BluePippin size selection system (Sage Science, Inc., Beverly, MA). Libraries were sequenced on the Pacific Biosciences RS II sequencer (PacBio, Menlo Park, CA) using a P4-C2 chemistry kit on ≥3 single-molecule real-time (SMRT) cells with a 180-min collection protocol. Sequencing reads were *de novo* assembled with the PacBio Hierarchical Genome Assembly Process 3 (HGAP3.0)/Quiver software package. The resulting assemblies were confirmed with optical maps generated with 30-fold coverage on the Argus Mapping Station, according to the OpGen protocol (OpGen, Inc, Gaithersburg, MD). The closed genomes were annotated using the NCBI Prokaryotic Genome Annotation Pipeline (PGAP) (http://www.ncbi.nlm.nih.gov/genome/annotation_prok/), and Ridom SeqSphere+ (Ridom GmbH, Münster, Germany) was used for *in silico* multilocus sequence type (MLST) analysis and to determine the presence of virulence genes.

The closed chromosomes of these STEC strains varied in size from 5.2 to 5.5 Mb, with an average G+C content of 50.7%, similar to those results found in other STEC strains (7). These STEC strains belonged to three sequence types (ST), and all carried one exceptionally large >200-kb virulence plasmid (8) (Table 1). Although these strains were isolated from foods and belong to serogroups that are rarely implicated in human disease (1), *in silico* analysis identified the enterotoxin-encoding genes *sta1* and *astA*, both associated with the development of diarrhea (9). Furthermore, two strains carried the highly HUS-associated *stx*_2a_ variant, one carried *stx*_1a_, and one carried *stx*_2g_; the role of stx2g in human pathogenicity remains to be elucidated (10). Additionally, these STEC strains carry genes encoding K88 fimbriae, long polar fimbriae (Lpf), the Iha adhesin, and/or the metalloprotease StcE (Table 1), which presumably enable these STEC strains to colonize the human gut (11–13). Moreover, all carry the glutamate decarboxylase gene *gad*, enabling these organisms to survive gastric acidity (14).

**TABLE 1:** Metadata of STEC strains and presence of virulence genes, as identified by *in silico* analysis

Strain	Serotype	Source	Size (bp)	G+C content (%)	ST	Virulence factors and other genetic features^a^											Accession no.
						*stx*	*eae*	*ehxA*	*iha*	*sta1*	*astA*	K88	*iss*	*gad*	*lpfA*	*stcE*	
CFSAN004178	O36:H14	Alfalfa sprouts	5,498,453	50.6	1176	2g	−		−		+		−	+	+		CP015229
			213,847	45.8				+	−	+	−	−				−	CP012498
CFSAN004179	O136:H16	Bagged lettuce	5,213,998	50.8	329	1a	−		+		−		−	+	+		CP013662
			242,187	47.0				+	−	+	+	+				+	CP012501
CFSAN004180	O168:H8^b^	Lettuce	5,286,558	50.7	718	2a	−		+		−		+	+	+		CP015228
			225,292	46.3				+	−	+	+	+				−	CP012500
CFSAN004181	O168:H8	Ground beef	5,233,459	50.8	718	2a	−		−		−		+	+	+		CP013663
			223,952	46.3				+	−	+	+	+				−	CP012499

a *stx*, Shiga toxin variant; *eae*, intimin; *iha*, IrgA homologue adhesin; *sta1*, heat-stable enterotoxin; *astA*, enteroaggregative heat-stable enterotoxin; K88, fimbriae; *iss*, increased serum survival; *gad*, glutamate decarboxylase; *lpfA*, long polar fimbriae; *stcE*, metalloprotease.

b H-type identified *in silico*, initially reported as O168:HNT.

Trace-back analysis is crucial during investigations of foodborne illness outbreaks. The data provided can aid in future efforts to identify the source of infection when matching clinical, food, and environmental isolates. The availability of complete genome sequences will further contribute to the ongoing investigation of genetic differences among various pathogenic *E. coli* strains.

### Accession number(s).

The closed and annotated chromosome and plasmid sequences were deposited in GenBank and are listed in Table 1.
